# Paraoxonase 1: The Lectin-Like Oxidized LDL Receptor Type I and Oxidative Stress in the Blood of Men with Type II Obesity

**DOI:** 10.1155/2019/6178017

**Published:** 2019-10-15

**Authors:** D. Kupczyk, R. Bilski, K. Sokołowski, M. Pawłowska, A. Woźniak, K. Szewczyk-Golec

**Affiliations:** ^1^Department of Medical Biology and Biochemistry, Ludwik Rydygier Collegium Medicum in Bydgoszcz, Nicolaus Copernicus University in Toruń, Bydgoszcz, Karłowicza 24 85-092, Poland; ^2^Specialized in Family Medicine Clinic Dr. Dariusz Górecki and Partners, Przesmyk 2/4 87-100 Toruń, Poland

## Abstract

**Objectives:**

Obesity has serious consequences such as the onset of metabolic syndrome, type 2 diabetes, atherosclerosis, or cardiovascular complications. The aim of this study was to evaluate the levels of paraoxonase 1 (PON1), lectin-like oxidized LDL receptor-1 (LOX-1), antioxidant enzymes (superoxide dismutase (SOD), catalase (CAT), and glutathione peroxidase (GPx)), and lipid peroxidation processes in the course of obesity.

**Methods:**

28 men took part in the experiment. Fourteen of them were obese; the control group consisted of 14 physically active men without obesity features. The concentrations of malondialdehyde (MDA), PON1, LOX-1, and tumor necrosis factor *α* (TNF*α*) as well as the activities of erythrocytic SOD, CAT, and GPx were determined in the study.

**Results:**

Statistically significant higher MDA, LOX-1, and TNF*α* levels were observed in obese subjects. Conversely, lower concentrations of PON1 in obese men were found.

**Conclusions:**

An imbalance in oxidation-reduction processes accompanies obesity. Moreover, inflammatory cytokines and atherosclerotic complications are involved in the obesity process. The obtained results suggest that the studied parameters may be independent prognostic markers preceding the development of cardiovascular and metabolic complications in people afflicted with type II obesity.

## 1. Introduction

The incidence of obesity, which is classified as chronic metabolic disease, shows an upward trend, especially in highly developed countries [1, 2]. Numerous epidemiological studies confirm the fact that the number of people with overweight and obesity is increasing all over the world. This problem affects both children and adults. As a consequence, obesity leads to the development of many systemic disorders, such as metabolic syndrome, type 2 diabetes, atherosclerosis, and cardiovascular complications [3–5]. The current criterion for assessing excessive body mass, recommended by the World Health Organization (WHO) and International Obesity Task Force (IOTF ), is the BMI (body mass index), calculated by dividing body mass (in kilograms) by the square of height (in meters). Obesity is diagnosed when this index is ≥30 kg/m^2^ [6]. The major factors leading to the development of simple obesity are poor dietary habits, concerning both quantity and quality of calories, and deficiency of physical activity. A smaller share is attributed to genetic factors, which include the polymorphism of following genes: fat mass and obesity-associated gene (FTO), commonly referred to as the “obesity gene” [7], and gene coding for peroxisome proliferator-activated receptor gamma (PPAR*γ*), resistin, and adiponectin [6]. In secondary obesity, etiopathology is more complex and may include drug-induced syndromes caused by glucocorticoids, central nervous system disorders, and endocrinopathies [8]. Obesity, especially of the central type, is an important component of metabolic syndrome, because it is the major factor responsible for the development of insulin resistance. The diagnosis of metabolic syndrome is made when carbohydrate and lipid metabolism disorders and hypertension coexist. It is insulin resistance that underlies the abovementioned disorders [9]. Therefore, criteria for the diagnosis of metabolic syndrome include increased HDL (high-density lipoprotein) cholesterol fraction, the presence of hypertriglyceridemia, elevated fasting blood glucose, the presence of hypertension, and waist circumference in men of ≥94 cm and in women ≥80 cm, which is an indicator of abdominal obesity [10]. Then, as a result of the accumulation of visceral fat, chronic inflammation develops due to an imbalance between the production of pro- and anti-inflammatory cytokines [6]. The atherosclerotic process, initiated by obesity, plays an important role in the pathogenesis of cardiovascular disease. The mechanism that underlies this process is the oxidative transformation of lipoproteins, leading to the formation of lipid peroxides. As a result of phagocytosis of oxidized lipoprotein molecules, foam cells are formed, contributing to the development of atherosclerotic lesions. Oxidative theory of atherosclerosis assumes that the process of atherogenesis is significantly influenced by modifications of low density lipoproteins (LDLs), which task is to supply cholesterol to cells [11]. The LDL oxidation process leads to the formation of various lipid fragments and protein lipoproteins. Therefore, lipid peroxidation products are formed, which can modify lipoprotein molecules, leading to their lipid overloading. If they are not recognized by the LDL receptor, they will continuously flow into the vessel wall. This is how oxidized low-density lipoproteins (OxLDLs) are formed when, after starting the “scavenger receptor” pathway, lipid peroxidation products lead to the modification of the protein part of LDL, namely, apolipoprotein B-100 (apoB-100) [12, 13]. OxLDLs are captured, inter alia, by the lectin-like receptor of oxidized LDL type I (LOX-1). LOX-1 is a 50 kD receptor belonging to the class E scavenger receptors [14]. In its structure, the common domain for C-type lectin receptors on NK (natural killer) cells is present [15]. LOX-1, after binding to the ligand, leads to the formation of multimers bound in the plasma lipid layer. The role of LOX-1 is to reduce smooth muscle cell proliferation and prevent platelet aggregation and monocyte chemotaxis. Therefore, the participation of this receptor in response to the occurrence and development of atherosclerotic lesions is indicated [16]. It is also worth noting that the major product of lipid peroxidation is malondialdehyde (MDA). Its concentration increases when the body develops the increased production of reactive oxygen species (ROS), which is an indicator of the disruption of the antioxidant barrier of the body [17]. A high-density lipoprotein fraction is associated with an enzyme that hydrolyzes ester bonds in the body—paraoxonase 1 (PON1). In the 90s, it was described as having antioxidant capabilities. It allowed the hypothesis that PON1 is responsible for the protection of LDLs against oxidation, which is important in the pathogenesis of atherosclerosis [18]. PON1 is a glycoprotein with a mass of 43-47 kDa, in the center of which there are two calcium ions, performing both the stabilizing and catalytic ion function. PON1 consists of 345 molecules of amino acids in which the N-terminal methionine is removed in the process of secretion and maturation. The hydrophobic signal sequence is necessary for paraoxonase 1 to be bound to the HDL particle. The activity of PON1 depends on the polymorphism of its gene [19]. The presence of glutamine at position 191 effectively protects LDL particles from oxidation, which provides protection against atherosclerosis. Due to the fact that OxLDLs are involved in the formation of foam cells and the destabilization of atherosclerotic plaque, have cytotoxic and procoagulant activity, and increase the expression of adhesion molecules in endothelial cells, the role of PON1 in their formation inhibition should be emphasized. When OxLDLs are formed and accumulated, PON1 is inactivated by reducing the number of free sulfhydryl groups of cysteine and by displacement of calcium ions by copper ions. The inactivation of PON1 also can be done by free radicals, especially hydrogen peroxide, which is the most reactive form of oxygen, formed during oxidative stress due to atherosclerotic lesions [20, 21]. At the moment, attention is paid to more and more factors and mechanisms predisposing to obesity or the appearance of atherosclerotic lesions. Although all the properties of paraoxonase 1 are not fully understood, it is known that this enzyme, by affecting the transformation of high-density lipoproteins, may have a protective function against the development of atherosclerosis, which is a consequence of obesity. Hence, in this study, an attempt was made to determine the concentration of this enzyme and its contribution to lipoprotein metabolism in the course of obesity.

## 2. Material and Methods

A total of 28 men participated in the experiment. In the study group, there were 14 men with simple obesity of the second degree. All patients received standard physical examinations and routine clinical laboratory tests. Body height of every person participating in the study was measured using a portable anthropometer. Height was measured without shoes, a hat, complicated hairstyles, or hair ornaments. Each subject stood upright, with his back on the height meter in such a way that the head, shoulders, buttocks and heels touched the device. The hands of the examined person were laid loosely along the body. The ear canal was in line with the cheekbone. Body mass was measured using a verified weight. Each tested person stood upright in the center of the weight, looked straight ahead, distributing the body weight evenly over both legs. The BMI value was obtained by the quotient of body mass in kg and squared body height in meters (kg/m^2^). Type II obesity was diagnosed when the BMI value was ≥35 kg/m^2^. Criteria for the inclusion and exclusion of the patients are presented in Table 1. All subjects provided informed consent to participate in the research. The study was approved by the Local Ethics Committee at Collegium Medicum, Nicolaus Copernicus University (Bydgoszcz, Poland). The control group consisted of 14 healthy, physically active men.  Table 2 provides the characteristics of the study participants.

Blood samples for biochemical tests were collected at 8:00 after overnight fasting from the cubital vein to the tubes without anticoagulant (3 mL) to obtain serum and to the lithium heparin tubes (3 mL) to obtain plasma and erythrocytes. Next, the collected samples were transported to the Department of Medical Biology and Biochemistry at the Collegium Medicum in Bydgoszcz of the Nicolaus Copernicus University in Toruń. All samples were centrifuged (6,000*g* for 10 min at 4°C). The serum or plasma were separated and stored at −80°C for further analysis. Subsequently, the erythrocytes were washed three times with a phosphate-buffered saline (PBS) solution at a ratio of 1 : 3 with a simultaneous centrifugation of the sample after each wash (6,000*g* for 10 min at 4°C). The washed red blood cells were mixed with a PBS solution to obtain erythrocytic suspension with 50% hematocrit index. The suspension was used to determine the parameters of oxidative stress. The malondialdehyde (MDA) and the activities of Zn,Cu-superoxide dismutase (SOD-1; EC 1.15.1.1), catalase (CAT; EC 1.11.1.6), and cytosolic glutathione peroxidase (GSH-Px; EC 1.11.1.9) were assayed by the methods described by Buege and Aust [22] in the modification of Esterbauer and Cheeseman [23], Paoletti and Mocali [24], Beers and Sizer [25], and Paglia and Valentine [26], respectively. The MDA level was read at 532 nm as a concentration of thiobarbituric acid reactive substances. SOD-1 activity was determined at 37°C by recording the increase in absorbance at 480 nm. One unit (U) of SOD-1 activity was defined as the amount of enzyme that inhibited the maximal rate of adrenaline auto-oxidation by 50%. CAT activity was recorded at 25°C by recording H_2_O_2_ decomposition measured at 240 nm. One Bergmeyer unit of this activity was defined as the amount of enzyme that decomposed 1 g of H_2_O_2_ per minute. The activity of GSH-Px was determined at 25°C by measuring the decrease in absorbance at 340 nm. The principle of the method for GPx activity measurement was based on the recording the decrease in absorbance of NADPH at 340 nm using H_2_O_2_ and reduced glutathione as substrates, GSH, yeast glutathione reductase, and NaN_3_ as a CAT inhibitor. One unit (U) of GSH-Px activity was defined as the amount of the enzyme that oxidized 1 *μ*mol of NADPH/minute. The hemoglobin concentration in the hemolysate was read at 540 nm after conversion into the cyanmethemoglobin form using a commercial reagent (BIOMED, Lublin, Poland).

To obtain serum, the blood samples were centrifuge for 5 min at 5000 × *g* and then transferred to the eppendorf tubes and frozen at −80°C. The serum thus prepared was stored for the determination of PON1, LOX-1, OxLDLs, and tumor necrosis factor *α* (TNF*α*) concentrations. The commercially available enzyme immune assay kits were used to estimate the serum concentrations of PON1 (human paraoxonase1 ELISA, BioVendor, Brno, Czech Republic), LOX-1 (human soluble lectin-like oxidized low-density lipoprotein receptor-1 (LOX-1) ELISA kit, AVISCERA Bioscience, Santa Clara, CA, USA), OxLDL (Ox-LDL ELISA kit, Immundiagnostik, Bensheim, Germany), and TNF*α* (human TNF*α* ELISA kit, Besancon, France), according to the manufacturers' instructions.

## 3. Statistical Analysis

Statistical analysis was performed using the STATISTICA computer program. Values of the parameters determined are expressed as the mean ± S.E. Statistical analysis included Student's *t*-test for the comparison of the study and control groups. The hypothesis of normal distribution was assessed by the Shapiro-Wilk test. In turn, the homogeneity of variances was verified using the Levene's test. *P* < 0.05 was considered statistically significant.

## 4. Results

The results of the parameters measured are presented in Table 3. The serum paraoxonase 1 concentration was lower, whereas the serum LOX-1 concentration was higher in the study group when compared with the control group. OxLDL level showed no significant difference between groups. The concentrations of TNF*α*, MDA in erythrocytes, and MDA in plasma were significantly higher in obese patients in comparison with healthy physically active subjects. SOD-1 and GPx activities showed no significant differences between the study and control groups. However, catalase activity was higher in the study group.

## 5. Discussion

Recently, interest in PON1 has focused on its antioxidant properties, instead of its role in phosphorylation of organic compounds. It turned out that PON1 is involved in the hydrolysis of compounds that cause atherosclerosis. The protective action of this enzyme in the process of atherosclerosis is more and more emphasized. In addition, PON1 activity can be modified by environmental factors and lifestyle. Physical activity is a protective factor against the development of cardiovascular diseases. This is confirmed by the conducted studies in which the activity of paraoxonase 1 was shown to be lower in people who have a sedentary lifestyle [27]. In addition, in these studies, it was found that regular physical exercise exerts a positive effect on the activity of this enzyme. In the presented study, a lower concentration of paraoxonase 1 in the group of obese men, in comparison with the slim and physically active subjects, was also found. It is currently believed that low PON1 activity is a risk factor for cardiovascular complications in people with obesity [28]. Abdominal obesity is one of the risk factors for the diagnosis of the metabolic syndrome according to the criteria adopted by the International Diabetes Federation (IDF) [29]. Numerous studies have shown a reduction in paraoxonase 1 activity in people with metabolic syndrome [30, 31].At present, more and more factors are being discussed that predispose early to the onset of atherosclerotic lesions and, consequently, to cardiovascular diseases. In this respect, attention is paid to the antiatherosclerotic action of high density lipoproteins and their contribution to the prevention of the oxidation of low density lipoproteins, and thus to provide protection against atherosclerotic lesions. Paraoxonase 1 plays a significant role in the conversion of the lipoproteins described above [20]. This is of great importance in the context of the studied men because second-degree obesity is a significant risk factor for cardiovascular disease. In the pathogenesis of atherosclerosis, lipid metabolism disorders play an important role. Lowered PON1 concentration in the blood is associated with an increased risk of this disease. The discovery of its biological role seems to be important for understanding the mechanisms of atherosclerosis. As a result of damage caused by atherosclerotic process, oxidation of LDL particles and the formation of so-called ox-LDLs occur. Then, oxidized LDLs are captured by both macrophages and receptors, namely, lectin-like LDL-type I receptor and scavenger receptors belonging to classes A and B. This metabolic pathway of LDLs plays a significant role in the formation of atherosclerotic lesions. It is confirmed by the overexpression of the LOX-1 receptor in cells affected by the atherosclerotic process [32]. Inflammatory cytokines, oxidative stress, and ox-LDLs can induce the expression of LOX-1. Diabetes mellitus is a condition, in which endothelial dysfunction, oxidative stress augmentation, and increased expression of adhesion molecules occur concurrently. In particular, type II diabetes correlates with an increased incidence of ischemic heart disease and cardiovascular complications. The development of obesity occurs, among others, due to disturbed carbohydrate metabolism. Obesity as a component of the metabolic syndrome is closely involved in the etiopathology of atherosclerosis and cardiovascular diseases. The relationship between LOX-1 and obesity is increasingly noticed. Chui et al. [33] showed a twofold increase in LOX-1 expression in obese mice compared to mice without obesity features. In our study, increased concentration of the LOX-1 was found in the group of obese men compared to the control group. Takanabe-Mori et al. [34] induced LOX-1 expression in obese mice on a high-fat diet and showed a positive correlation with body mass. In addition, Rasouli et al. observed a positive correlation between LOX-1 expression and human BMI. The abovementioned studies confirm the participation of LOX-1 in obesity and the development of diabetes and inflammatory changes. OxLDL is the marker of oxidative stress and is considered to be a prognostic marker for the progression of subclinical atherosclerosis. In our study, there was no significant difference in this parameter between the study and control groups [35]. On the contrary, in the study of Van Guilder et al. [36], significantly higher plasma concentrations of OxLDLs in obese adults in comparison with normal-weight controls were shown. The activation of the inflammatory process is responsible for the formation of atherosclerotic changes in obesity, which can be confirmed by an increase in the level of pro-inflammatory cytokines, including tumor necrosis factor *α* [37]. In our study, a higher concentration of this cytokine was found in the group of obese men compared to the control group. Special attention is paid to the role of oxidative stress in the development of obesity, diabetes, hypertension, dyslipidemia, or atherosclerosis [38]. Under normal conditions, reactive oxygen species are released in the body in safe amounts. They play the role of mediators and regulators of cell processes. Excessive production of ROS results in pathological changes in the function and structure of proteins, cell membrane lipids, and DNA damage. As a result of the phenomenon of oxidative stress, lipid peroxidation occurs. This is the process of oxidation of unsaturated fatty acids constituting phospholipids of cell membranes. It leads to the formation of lipid peroxides. The main product of these reactions is malondialdehyde, the compound which concentration increases under augmented oxidative stress, leading to modifications in the permeability of cell membranes [39]. In this study, higher levels of MDA both in plasma and erythrocytes were observed in the group of obese men in comparison to the control group, indicating the higher intensity of lipid peroxidation in obesity. Similarly, increased levels of MDA in patients with obesity have been demonstrated in other studies [40, 41]. Analogous results were presented by Agrawal and Singh [42]. They demonstrated higher levels of MDA concentration in patients with grade 1, 2, or 3 obesity in comparison with the control group of healthy subjects with body mass index of 19-25. In the research of Hamma et al. [43], higher MDA concentration in patients with BMI 25 and above in comparison with control group with BMI < 25 was also observed. The same results were presented by Singh K. and Singh S. [44]. In their study, the MDA concentration was higher in the group of North West obese Indians than that in healthy male subjects. Elevated level of reactive oxygen species and decrease antioxidant enzymes are the markers of oxidative stress in obese patients. In the study of Agrawal et al. [42], there was a significantly lower activity of SOD in comparison with the control group. Similar results were obtained by Tinahones et al. [45]. In contrast, in our study, there were no statistically significant differences in erythrocytic SOD-1 activity. Hamma et al. [43] also showed no significant difference in SOD between the compared BMI categories. CAT and GPx also belong to important biochemical markers of oxidative stress in obese patients. Singh K. and Singh S. [44] showed lower levels of CAT and GPx activities in obese patients in comparison with normal, healthy male subjects. Hamma et al. [43] showed no significant difference in GPx activity between the BMI categories. In our study, there was also no significant difference in GPx activity between the study and control groups. However, significantly higher activity of CAT in obese patients in comparison with healthy control subjects was demonstrated. It could be considered as a compensatory mechanism for increased production of reactive oxygen species in the course of obesity. Our results show that the reduced concentration of PON1 in the blood of the test subjects is associated with the increased risk of atherosclerosis, because it plays a major role in this pathogenesis lipoprotein metabolic disorders. PON1 plays an important role in lipoprotein conversion, which, if the concentration of this enzyme is disturbed, is abnormal.

The results show the participation of both PON1 and LOX in the pathogenesis of cardiovascular diseases play a significant role in men with type II obesity. The increased oxidative stress was also assessed in this group of patients. The major limitation of our study is the number of the subjects studied. Our results indicate that the research regarding this particular factors should be expanded with a larger study group and different types of obesities.

## 6. Conclusions

Obesity is an increasingly common phenomenon in society that causes serious complications in the form of metabolic syndrome, diabetes, or cardiovascular diseases. That is why information is increasingly sought regarding the mechanisms of pathogenesis of this phenomenon and thus the possibility of preventing its severe complications. In the presented study, a decrease in PON1 concentration and an increase in MDA in obese men were found suggesting a disturbance of the pro/antioxidant balance of the body. In atherosclerosis, the lipoprotein metabolism disorder occurs. PON1 is an important enzyme in the conversion of lipoproteins. Therefore, the decreased concentration of PON1 plays an important role in pathogenesis of atherosclerosis. The increase in MDA level suggests intensification of lipid peroxidation process in obese patients. The observed higher CAT activity points to the compensatory mechanism in simple obesity against augmented ROS production. The study showed that obesity is an independent risk factor in the production of reactive oxygen species which can lead to cell damage. Increased TNF*α* concentration strongly indicates the involvement of inflammatory process in obesity. Higher LOX-1 concentration may be explained by the occurrence of pathological processes leading to the atherosclerotic complications in the group of men with type II obesity. Moreover, our observations suggest that LOX-1 plays an important role in adipose inflammation. It could be concluded that the studied parameters may be independent prognostic markers preceding the development of cardiovascular and metabolic complications in people afflicted with type II obesity.

## Figures and Tables

**Table 1:** Inclusion and exclusion criteria for participation in the study.

Groups of patients	Criteria for inclusion	Criteria for exclusion
Healthy subjects	Gender: male	Addiction to alcohol/tobacco
	Physically active lifestyle	Diabetes mellitus or other conditions of known free radical etiology
	Informed consent for participation in the study	
	BMI normal	

Obese patients	Gender: male	Addiction to alcohol/tobacco
	Obesity of the second degree	Metabolic syndrome, diabetes mellitus, or other conditions of known free radical etiology
	Glucose level < 100 mg/dl	Hypertension

**Table 2:** Characteristics of study participants.

Parameter	Study group		Control group	
	Mean	SE	Mean	SE
Age [years]	35.43	4.04	33.14	4.03
Height [m]	1.81	0.02	1.80	0.02
Body mass [kg]	126.59	5.05	79.29	2.71
BMI [kg/m^2^]	38.46	1.30	24.44	0.52

**Table 3:** Comparison of the mean values of the determined parameters in the study and control group.

Parameter	Study group		Control group		*P*
	Mean	Standard error	Mean	Standard error	
PON1 [10 pg/ml]	10.18	0.86	17.57	0.12	<0.01
LOX-1 [pg/ml]	119.0	17.54	68.71	16.47	<0.01
TNF*α* [pg/ml]	20.60	14.22	10.24	5.81	<0.01
Erythrocytic MDA [nmol/gHb]	31.07	4.26	17.95	1.96	<0.01
Plasma MDA [nmol/ml]	0.46	0.02	0.34	0.01	<0.05
SOD-1 [U/gHb	728.22	20.90	728.13	20.72	NS
CAT [10^4^ × IU/gHb]	68.50	2.53	57.44	2.14	<0.05
GPx [U/gHb]	6.75	1.46	7.48	1.47	NS
OxLDL [ng/ml]	172.18	29.12	170.82	13.18	NS

NS: not significant.

## Data Availability

The data used to support the findings of this study are included within the article.

## References

[1] James 
W. P. T. (2008). The epidemiology of obesity: the size of the problem. *Journal of Internal Medicine*.

[2] WHO Obesity and overweight. https://www.who.int/mediacentre/factsheets.

[3] Eckel R. H., Grundy S. M., Zimmet P. Z. (2005). The metabolic syndrome. *The Lancet*.

[4] Cook S., Weitzman M., Auinger P., Nguyen M., Dietz W. H. (2003). Prevalence of a metabolic syndrome phenotype in adolescents: findings from the third National Health and Nutrition Examination Survey, 1988–1994. *Archives of Pediatrics & Adolescent Medicine*.

[5] Małecka-Tendera E., Erhardt E., Molnár D. (2007). Type 2 diabetes mellitus in European children and adolescents. *Acta Paediatrica*.

[6] Jung A. (2014). Obesity – a lifestyle disease. *Pediatria i Medycyna Rodzinna*.

[7] Kalantari N., Doaei S., Keshavarz-Mohammadi N., Gholamalizadeh M., Pazan N. (2016). Review of studies on the fat mass and obesity-associated (FTO) gene interactions with environmental factors affecting on obesity and its impact on lifestyle interventions. *ARYA Atherosclerosis*.

[8] Bujalska I. J., Kumar S., Stewart P. M. (1997). Does central obesity reflect "Cushing's disease of the omentum"?. *The Lancet*.

[9] Reaven G. M. (1988). Banting lecture 1988. Role of insulin resistance in human disease. *Diabetes*.

[10] Alberti K. G. M. M., Zimmet P., Shaw J. (2006). Metabolic syndrome-a new world-wide definition. A Consensus Statement from the International Diabetes Federation. *Diabetic Medicine*.

[11] Strzyżewski K., Pioruńska-Stolzman M., Majewski W. (2008). Ocena peroksydacji lipidów i stężenia wybranych antyoksydantów w surowicy pacjentów z miażdżycowym niedokrwieniem kończyn dolnych. *Nowiny Lekarskie*.

[12] Nording H., Giesser A., Patzelt J. (2016). Platelet bound oxLDL shows an inverse correlation with plasma anaphylatoxin C5a in patients with coronary artery disease. *Platelets*.

[13] Wang W., Wang D., Kong C. (2019). eNOS S-nitrosylation mediated OxLDL-induced endothelial dysfunction via increasing the interaction of eNOS with *β*‑catenin. *Biochimica et Biophysica Acta (BBA) - Molecular Basis of Disease*.

[14] Mehta J. L., Li D. (2002). Identification, regulation and function of a novel lectin-like oxidized low- density lipoprotein receptor. *Journal of the American College of Cardiology*.

[15] Chen M., Narumiya S., Masaki T., Sawamura T. (2001). Conserved C-terminal residues within the lectin-like domain of LOX-1 are essential for oxidized low-density-lipoprotein binding. *Biochemical Journal*.

[16] Chen M., Masaki T., Sawamura T. (2002). LOX-1, the receptor for oxidized low-density lipoprotein identified from endothelial cells: implications in endothelial dysfunction and atherosclerosis. *Pharmacology & Therapeutics*.

[17] Kar K., Bhattacharyya A., Paria B. (2018). Elevated MDA level correlates with insulin resistance in prediabetes. *Journal of Clinical and Diagnostic Research*.

[18] Göçmen A. Y., Gűműşlű S., Gűnaydin I., Semiz E. (2012). Paraoxonase-1 activity and the levels of lipids and lipid peroxidation markers in arterial versus venous blood samples in coronary angiography patients. *Advances in Interventional Cardiology*.

[19] Gajewski P., Tomaniak M., Filipiak K. J. (2015). Paraoksonaza 1—co o niej obecnie wiadomo?. *Folia Cardiologica*.

[20] Durrington P. N., Mackness B., Mackness M. I. (2001). Paraoxonase and atherosclerosis. *Arteriosclerosis, Thrombosis, and Vascular Biology*.

[21] Aviram M., Billecke S., Sorenson R. (1998). Paraoxonase active site required for protection against LDL oxidation involves its free sulfhydryl group and is different from that required for its arylesterase/paraoxonase Activities. *Arteriosclerosis, Thrombosis, and Vascular Biology*.

[22] Buege J. A., Aust S. D. (1978). [30] Microsomal lipid peroxidation. *Methods in Enzymology*.

[23] Esterbauer H., Cheeseman K. H. (1990). [42] Determination of aldehydic lipid peroxidation products: Malonaldehyde and 4-hydroxynonenal. *Methods in Enzymology*.

[24] Paoletti F., Mocali A. (1990). [18] Determination of superoxide dismutase activity by purely chemical system based on NAD(P)H oOxidation. *Methods in Enzymology*.

[25] Beers R. F., Sizer I. W. (1952). A spectrophotometric method for measuring the breakdown of hydrogen peroxide by catalase. *Journal of Biological Chemistry*.

[26] Paglia D. E., Valentine W. N. (1967). Studies on the quantitative and qualitative characterization of erythrocyte glutathione peroxidase. *Journal of Laboratory and Clinical Medicine*.

[27] Otocka-Kmiecik A., Orłowska-Majdak M. (2009). The role of genetic (PON1 polymorphism) and environmental factors, especially physical activity, in antioxidant function of paraoxonase. *Postępy Higieny i Medycyny Doświadczalnej*.

[28] Fülöp P., Harangi M., Seres I., Paragh G. (2016). Paraoxonase-1 and adipokines: potential links between obesity and atherosclerosis. *Chemico-Biological Interactions*.

[29] Drzycimska-Tatka B., Drab-Rybczyńska A., Kasprzak J. (2011). Zespół metaboliczny- epidemia XXI wieku. *Hygeia Public Health*.

[30] Vavrova L., Kodydkova J., Zeman M. (2013). Altered activities of antioxidant enzymes in patients with metabolic syndrome. *Obesity Facts*.

[31] Hashemi M., Kordi-Tamandani D. M., Sharifi N. (2011). Serum paraoxonase and arylesterase activities in metabolic syndrome in Zahedan, southeast Iran. *European Journal of Endocrinology*.

[32] Kuge Y., Kume N., Ishino S. (2008). Prominent lectin-like oxidized low density lipoprotein (LDL) receptor-1 (LOX-1) expression in atherosclerotic lesions is associated with tissue factor expression and apoptosis in hypercholesterolemic rabbits. *Biological & Pharmaceutical Bulletin*.

[33] Chui P. C., Guan H. P., Lehrke M., Lazar M. A. (2005). PPAR*γ* regulates adipocyte cholesterol metabolism via oxidized LDL receptor 1. *Journal of Clinical Investigation*.

[34] Takanabe-Mori 
R., Ono K., Sowa N. (2010). Lectin-like oxidized low-density lipoprotein receptor-1 is required for the adipose tissue expression of proinflammatory cytokines in high-fat diet- induced obese mice. *Biochemical and Biophysical Research Communications*.

[35] Rasouli N., Yao-Borengasser A., Varma V. (2009). Association of scavenger receptors in adipose tissue with insulin resistance in nondiabetic humans. *Arteriosclerosis, Thrombosis, and Vascular Biology*.

[36] Van Guilder G. P., Hoetzer G. L., Greiner J. J., Stauffer B. L., DeSouza C. A. (2006). Influence of metabolic syndrome on biomarkers of oxidative stress and inflammation in obese adults. *Obesity*.

[37] Tamakoshi K., Yatsuya H., Kondot E. (2003). The metabolic syndrome is associated with elevated circulating C-reactive protein in healthy reference range, a systemic low-grade inflammatory state. *International Journal of Obesity*.

[38] Matsuda M., Shimomura L. (2013). Increased oxidative stress in obesity: implications for metabolic syndrome, diabetes, hypertension, dyslipidemia, atherosclerosis, and cancer. *Obesity Research and Clinical Practice*.

[39] Kulbacka J., Saczko J., Chwiłkowska A. (2009). Stres oksydacyjny w procesach uszkodzenia komórek. *Polish Medical Journal*.

[40] Van Gaal L. F., Vertommen J., De Leeuw I. H. (1998). The *in vitro* oxidizability of lipoprotein particles in obese and non-obese subjects. *Atherosclerosis*.

[41] Skalicky J., Muzakova V., Kandar R., Meloun M., Rousar T., Palicka V. (2008). Evaluation of oxidative stress and inflammation in obese adults with metabolic syndrome. *Clinical Chemistry and Laboratory Medicine*.

[42] Agrawal N., Singh S. K. (2017). Correlation of oxidative stress parameters with various grades of obesity. *International Journal of Medical and Health Research*.

[43] Hamma S. A., Fergani I., Lakehal A., Abadi N., Benlatreche C. (2015). Oxidative stress in Algerian adults obesity. *Journal of Metabolic Syndrome*.

[44] Singh K., Singh S. (2015). Comparative study on malondialdehyde and certain antioxidants in North West obese Indians. *Journal of Cardiovascular Disease Research*.

[45] Tinahones F. J., Murri-Pierri M., Garrido-Sánchez L. (2008). Oxidative stress in severely obese persons is greater in those with insulin resistance. *Obesity*.

